# Supplementary material for: Mycobacterium leprae transmission characteristics during the declining stages of leprosy incidence: A systematic review
Source: PLoS Negl Trop Dis. 2021 May 26;15(5):e0009436. doi: 10.1371/journal.pntd.0009436 (PMC8186771; doi:10.1371/journal.pntd.0009436)
Supplement: Attachment — Submitted filename: Response letter PLOS NTDs_210421.pdf [file pntd.0009436.s003.pdf]

Erasmus MC
University Medical Center Rotterdam

Department of Public Health

Direct dial 010 – 703 84 73
Fax number
Internal postal address Na-2310
E-mail t.hambridge@erasmusmc.nl
Our reference
Date 21 April 2020

Postal address
P.O. box 2040
3000 CA Rotterdam, NL

Visiting address
Wytemaweg 80
3015 CN Rotterdam, NL

Car Park
Wytemaweg 12
3015 CN Rotterdam

Dear PLOS Neglected Tropical Diseases Editors,

We are writing to resubmit our manuscript entitled "*Mycobacterium leprae* transmission characteristics during the declining stages of leprosy incidence: a systematic review" with significant revisions. Please see below our responses to the review comments and a description of the changes we have made in the manuscript (lines correspond to clean version):

Methods

Reviewer #1: No. This is a descriptive study which offers little new insight and little analysis.

We thank the reviewer for their feedback. The purpose of this systematic literature review was to shed light on the question as to whether transmission of *M. leprae* is driven mainly by undiagnosed and untreated new leprosy cases in the community, or by incompletely treated or relapsing old cases. This was a recent point of discussion within the leprosy research community and has important policy implications for leprosy control. Due to the nature of the case data collected, we were unable to perform a meta-analysis and decided to proceed with a comprehensive descriptive analysis.

The International Federation of Anti-Leprosy Associations (ILEP) Technical Commission requested this descriptive study be conducted to examine this dilemma. This was first proposed in September 2019, with the results presented and discussed in October 2020 (minutes from both meetings enclosed). We have made the goal of the review more explicit in the introduction and abstract.

Reviewer #2: NA

Reviewer #3: This a systematic review of published literature of leprosy case characteristics in low endemic countries. The authors used standard search methodology and their study inclusion and exclusion criteria seem appropriate.

Results

Reviewer #1: Excellent narrative descriptions of leprosy in different countries, but little to no analysis or projection of trends

Our systematic review had specific research questions that did not include examining change in incidence over time or making future projections, but rather to describe the control measures implemented in each setting and use patient data to describe case characteristics. Trends of leprosy case detection were reflected in both the introduction (line 80) and discussion (line 975) with reference to Meima A, et al. (2004).

Reviewer #2: NA

Reviewer #3: Yes

<u>Conclusions</u>

Reviewer #1: The primary conclusion of the study was that 'imported leprosy cases seem to have little impact on the endemic population, and they do not represent a large reservoir for ongoing transmission. This seems obvious from the start and no new analysis is included

We stated in the discussion (line 905) – 'there is no evidence to suggest that an increase in foreign born leprosy cases arriving from high endemic areas contribute to a noticeable rise in local transmission.'. However, this was not a primary conclusion of the study. We also disagree that this is an obvious conclusion, especially given the high number of imported MB cases reported in a range of global settings, including Canada, Italy, Spain, New Zealand, Taiwan (Republic of China) and the United States. Moreover, migration patterns have changed considerably over recent decades, necessitating a new estimation of whether there is transmission by foreign born cases. Nevertheless, we have made it clearer in the methodology that demonstrating these types of associations was not an objective of the study (line 203).

Reviewer #2: NA

Reviewer #3: Yes

<u>Summary and General Comments</u>

Reviewer #1: The authors present here a literature review concerning leprosy in low endemic areas with the notion that "As leprosy incidence begins to decline, characteristics of new cases shifts away from those observed in highly endemic areas, revealing potentially important insights into possible ongoing sources of transmission." They performed an exhaustive literature review covering 22 countries where they suggest 48% of the cases were imported, 64% were multibacillary and 18% had a family history. They conclude that "there was no indication that the [new] cases described here led to a rise in new secondary cases, suggesting that they do not represent a large ongoing source of human-to-human transmission."

Of course, these general observations are not new, and given the fact the countries reviewed are lowly-endemic, it seems rather obvious that the relative few endemic or imported cases reviewed would not represent a 'large ongoing source of human-to-

human transmission'. While they reiterate that imported cases seem to have little impact on endemic disease rates, they make no attempt to describe what source may sustain low level endemic transmission (other than perhaps some family relationships in up to 18% of cases), or address the classic irony that leprosy appears to have spread around the world by colonization, trade and adventurism; but in modern times introduction from foreign sources seems to have little impact.

A major difficulty comes in discerning where a case may have acquired their infection. Typically, if a patient reports having traveled or lived abroad at all, programs tend to classify their source to be foreign and also having been acquired abroad. However, the actual voracity of this assumption is actually unknown. Some programs require a diagnosis within 5, 7, or 10 years of the foreign exposure. Others may consider anytime abroad, even if only for short-term travel. The subtlety of discerning an imported from endemic case is not elaborated or considered. How do you know if an infection was acquired abroad?

The paper includes a large number of figures and tables which are not really utilized analytically. We see that the age, gender, disease-type and ratio of imported:autochthonous cases and relapse varies widely among the countries reviewed. While these figures are a compact way to annotate the individual country narratives, they really don't reveal anything about how these 'characteristics' have changed over time. In addition, these wide variations are likely impacted by the highly variable number of cases reviewed in different countries. Unfortunately, no statistical analysis was used to smooth the ratios or project some trends in the characteristics. Curiously, MDT and BCG vaccinations were highlighted as major contributing factors in achieving control of leprosy in many programs, but again there is no substantive analysis of these 'characteristics' between the low-endemic countries or between them and other persistently high endemic areas.

In recent times the role of non-human reservoirs of M. leprae has gained notoriety in the literature. The importance of these reservoirs in sustaining leprosy is not yet confirmed. The authors do note that armadillos are associated with 64% of the cases in the United States. Does the persistence of leprosy in low endemic areas suggest there might be other non-human reservoirs, especially given the fact that imported cases do not seem to be contributing greatly to ongoing human-to-human transmission? In addition, recent literature suggests armadillos in South America also may be involved in zoonotic transmission. When one major source of infection comes under control, other lessor sources rise in importance. Does persistence of infection in low-endemic areas suggest sources other than human-to-human likely play a role?

We agree with the reviewer that we should elaborate on other sources that may sustain low level endemic transmission outside of those with a family history and other suspected sources detailed in the case records. This has now been added to the discussion (lines 942 – 956), including comments on potential environmental reservoirs or zoonotic spread.

Regarding where a case may have acquired their infection, we agree with the reviewer. This cannot be established with absolute certainty. The definition of an imported case varied in our

study, but the reason for classification as suspected non-autochthonous was usually described in the paper (e.g. the individual had recently immigrated from a high-endemic area). The difficulties in determining a patient's exact history were also highlighted in the discussion section (line 900) and we have further described this uncertainty (line 903).

Due to the nature of the individual case data sampled it was not possible to perform any meaningful time trend analyses or make projections for individual countries. Performing an analysis of pooled case data would also not be suitable given the broad range of different contexts and timeframes studied. Instead, the aim was to make a single assessment of the leprosy case characteristics over the specified period of incidence decline. We have stated this as a limitation (line 995).

Reviewer #2: The authors performed a systematic review for the literatures of leprosy and summarized the transmission characteristics of mycobacterium leprae during the low incidence of leprosy. The paper has public health implications because it provides different measures for prevention and control of leprosy.

Major comments:

1) WHO globle leprosy programme will report chracteristics of newly onset leprosy cases in each country. The data is reported by the national leprosy management department. The author should analyze the similarities and differences between the official data and the literature based data. Limitation of the literature based data should also be highlighted.

2) The country description part is too lengthy. Those data should be better integrated and analyzed.

3) Did the author have the whole list of 105 studies involved in this analysis? It should be put at the supplementary file.

4) Taiwan is an indispensable part of China and has never been a country. The author should revised current statement.

We thank the reviewer for their feedback. Please our responses to each point below:

1) We agree with the reviewer and this has been highlighted in the discussion (line 991).

2) We have cut down the text for the country descriptions and removed data overlapping with the quantitative results section.

3) We have created a full list of the 105 peer-reviewed studies used to leprosy extract case data. This can be found in Supporting Information 2 (S2) and contains author, title, country, date of publication and the number of cases collected.

4) We thank the reviewer for highlighting this and now refer to Taiwan (Republic of China) throughout the manuscript.

Reviewer #3: In the introduction the authors should mention that leprosy can be caused by M. leprae and M. lepromatosis.

It may be helpful if the authors mention number of new cases in 2019 (when available) for the countries included in this review. For most countries this is now available in WHO database.

In the discussion the authors may emphasize the importance of access to health care and continued surveillance in rural areas for leprosy control. Although the data for urban vs rural cases were not available for all the reviewed low endemic countries, but countries like Iran, Morocco, Norway & Portugal showed the importance of surveillance in rural areas which for various reasons can harbor a more conducive environment for leprosy transmission than the urban areas.

We thank the reviewer for their feedback. *M. lepromatosis* has been added to the introduction as a cause of leprosy (line 64). For the countries included in this study, we have specified the 2019 new case numbers from latest WHO report where available. We agree with the reviewer's final point and have highlighted this in the discussion (line 1002).

Minor comments

Reviewer #1:
Line 1008: incorrectly suggests that humans and armadillos share a single genotype of M. leprae

We agree with the reviewer and have changed this sentence (line 950) to: 'A zoonotic transmission pathway from exposure to armadillos has been proposed, with human patients from a previous study in southeastern United States shown to be infected with the same armadillo-associated *M. leprae* genotype.'

Line 1009: rather obscure citation. Should cite the original articles.

We have now cited the original article - Sharma, R., et al. 2015

Line 1043: should include distinguishing endemic/imported as a major limitation.

This has been added as a limitation (line 987).

Reviewer #2:
1) Methods: Please clarify the range set of published date of the literature when conducting the literature search.

2) Methods: The authors included "the studies from countries or regions with less than one new leprosy case detected per 100,000 population", however, the incidence of leprosy in each country varies from year to year. Did the authors use the incidence of the specific year?

3) Results: As for the autochthonous cases reported, the detailed transmission route of these cases should be explained such as relapse, travelling to high endemic areas, or contact with animals like armadillos?

We thank the reviewer for their feedback. Please our responses to each point below:

1) We have added the publication date to the table in Supporting Information 2 (S2) for all 105 studies included in the quantitative analysis.

2) We have specified in the methodology section (line 166) that all countries or regions included had less than one new leprosy case detected per 100,000 population in the final year of the timeframe from which they were collected.

3) These details were described for autochthonous cases in the narrative descriptions where available. For example:

- Canadian male who's only travel was to Florida and *M. leprae* isolate from his biopsy was identified as the armadillo-associated genotype 3I-2-v1.
- A rare case of documented patient-to-surgeon transmission of *M. leprae* in Germany.
- Male diagnosed with LL in the United Kingdom, returning after 40 years in the tropics. Two young adults who were contacts later found to have raised antibodies to *M. leprae* and were subsequently given 6 months chemoprophylaxis with rifampicin

Reviewer #3:
None

We thank the members of the PLOS Neglected Tropical Diseases (NTDs) editorial board and independent reviewers for taking the time to provide feedback. Please don't hesitate to contact us if you require any additional information.

Sincerely,

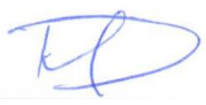

Thomas Hambridge
PhD Candidate
Erasmus MC, The Netherlands
