# Supplementary material for: Mycobacterium leprae transmission characteristics during the declining stages of leprosy incidence: A systematic review
Source: PLoS Negl Trop Dis. 2021 May 26;15(5):e0009436. doi: 10.1371/journal.pntd.0009436 (PMC8186771; doi:10.1371/journal.pntd.0009436)
Supplement: S1 List of Peer-reviewed Studies with Case Data — (DOCX) [file pntd.0009436.s002.docx]

**S1. List of Peer-reviewed Studies Containing Individual Case Data**

| **Author** | **Title** | **Country** | **Publication date** | **Cases** |
| --- | --- | --- | --- | --- |
| McKay, C., et al.^27^ | A case series of leprosy in New South Wales. | Australia | 2012 | 3 |
| McIver, L. J., et al.^23^ | Acute glomerulonephritis in a child with multidrug-resistant tuberculosis and multibacillary leprosy. | Australia | 2011 | 1 |
| Manifold, R., & Marshman, G.^24^ | Leprosy: Not always an easy diagnosis and often a management challenge. | Australia | 2009 | 2 |
| Edwards, L. J., et al.^21^ | Detection of *Mycobacterium leprae* by PCR Testing of sputa from a patient with pulmonary cryptococcus coinfection in Northern Australia. | Australia | 2014 | 1 |
| Coates, E.^25^ | Leprosy in Australia: Where are we now? | Australia | 2012 | 1 |
| Charlton, O. A., et al.^22^ | Leprosy: An ancient disease that still threatens global health. | Australia | 2017 | 1 |
| Barkla, S., & Modi, S.^26^ | Lepromatous leprosy: A rare presentation in Australia. | Australia | 2013 | 1 |
| Archibald, H., et al.^19^ | Locally acquired Hansen's disease in North Queensland. | Australia | 1999 | 1 |
| Saber, M., et al.^32^ | Canadian case report of erythema nodosum leprosum successfully treated with prednisone and thalidomide. | Canada | 2010 | 1 |
| Bonnar, P. E., et al.^31^ | Leprosy in nonimmigrant Canadian man without travel outside North America, 2014. | Canada | 2018 | 1 |
| Boggild, A. K., et al.^20^ | Leprosy in Toronto: an analysis of 184 imported cases. | Canada | 2004 | 184 |
| Chen, S., et al.^39^ | Rapid survey on case detection of leprosy in a low endemic situation, Zhucheng County, Shandong Province, the People's Republic of China. | China | 2007 | 7 |
| Chen, S., et al.^40^ | Should household contact examination in a low endemic situation of leprosy continue? | China | 2003 | 547 |
| Chu, T., et al.^35^ | Comprehensive measures succeeded in improving early detection of leprosy cases in post-elimination era: Experience from Shandong province, China. | China | 2020 | 231 |
| Schmiedel, S., et al.^46^ | A Thai patient with generalised inflammatory skin disease 18 years after migration to Europe. | Germany | 2006 | 1 |
| Saar, M., et al.^44^ | Report on an unusual case of leprosy from Germany: just an exception of the rule? | Germany | 2019 | 1 |
| Koch, A., et al.^43^ | Leprosy: Two case reports from Dresden, Germany. | Germany | 2006 | 2 |
| Biedermann, T., et al.^48^ | Leprosy type 1 reaction as the first manifestation of borderline lepromatous leprosy in a young native German. | Germany | 1997 | 1 |
| Becker, S. L., et al.^47^ | Multibacillary leprosy in a migrant from Afghanistan: A disease not to be forgotten. | Germany | 2017 | 1 |
| Achilles, E. G., et al.^45^ | Leprosy accidentally transmitted from a patient to a surgeon in a nonendemic area. | Germany | 2004 | 2 |
| Golfurushan, F., et al.^49^ | Leprosy in Iran: an analysis of 195 cases from 1994-2009 | Iran | 2011 | 195 |
| Farshchian, M., & Kheirandish, A.^50^ | Clinico-pathological study of 12 cases of patients with leprosy admitted to Sina Hospital, Hamadan, Iran, from 1991 to 2000. | Iran | 2004 | 12 |
| Simeoni, S., et al.^54^ | Leprosy initially misdiagnosed as sarcoidosis, adult-onset still disease, or autoinflammatory disease. | Italy | 2011 | 1 |
| Riccò, M., et al.^52^ | Epidemiology of leprosy in Italy (1920-2019): A comprehensive review on existing data. | Italy | 2019 | 14 |
| Massone, C., et al.^57^ | Histopathologic diagnosis of leprosy in a nonendemic area. | Italy | 2010 | 3 |
| Massone, C., et al.^56^ | Borderline lepromatous leprosy in an Italian man. | Italy | 2013 | 1 |
| Marotta, M., et al.^55^ | A case of imported leprosy in Italy: Implications for surveillance by Public Health Services of Local Health Authorities. | Italy | 2019 | 1 |
| Maritati, M., & Contini, C.^58^ | A Case of Leprosy in Italy: A Multifaceted Disease Which Continues to Challenge Medical Doctors. | Italy | 2016 | 1 |
| Liguori, R., et al.^59^ | An inflammatory myopathy unmasks a case of leprosy in an Italian patient. | Italy | 2015 | 1 |
| Fiallo, P., et al.^61^ | Leprosy in an Italian tourist visiting the tropics. | Italy | 1993 | 1 |
| Cusini, M., et al.^60^ | A case of autochthonous leprosy in an elderly Italian patient leaving in Milan province with peculiar clinical presentation. | Italy | 2017 | 1 |
| Abdelmaksoud, A., et al.^53^ | Topical 1% propranolol in liposomal gel: A new adjuvant tool for chronic leprosy ulcers. | Italy | 2018 | 3 |
| Ozaki, M., & Tomoda, M.^71^ | Conjugal leprosy infection in Japan - Case report and review. | Japan | 2012 | 2 |
| Noda, M., et al.^72^ | Leprosy with spontaneous type 1 lepra reaction as an initial cutaneous manifestation: a case from Japan. | Japan | 2018 | 1 |
| Namisato, M., et al.^65^ | Uveitis seen in the long clinical course of leprosy. | Japan | 2002 | 7 |
| Miyagi, H., et al.^64^ | Case of chromoblastomycosis appearing in an Okinawa patient with a medical history of Hansen's disease. | Japan | 2008 | 1 |
| Matsuura, H., et al.^66^ | An imported case of leprosy in a trainee from Indonesia to Japan. | Japan | 2017 | 1 |
| Matsuoka, M.,et al.^68^ | A second case of multidrug-resistant *Mycobacterium leprae* isolated from a Japanese patient with relapsed lepromatous leprosy. | Japan | 2003 | 1 |
| Koike, H., et al.^70^ | The Wide Range of clinical manifestations in leprous neuropathy: Two Case Reports. | Japan | 2011 | 2 |
| Kiriyama, I., et al.^69^ | Prostate cancer in patients with Hansen's disease. | Japan | 2003 | 3 |
| Ishii, N., et al.^67^ | Two sisters with leprosy. | Japan | 1993 | 2 |
| Toweir, A. A., & Chaudhary, R. C.^73^ | Review of leprosy cases in Benghazi, Libyan Arab Jamahiriya, 1994-1998 | Libya | 2000 | 54 |
| Jacobson, R. R., & Gatt, P.^74^ | Can leprosy be eradicated with chemotherapy? An evaluation of the Malta Leprosy Eradication Project. | Malta | 2008 | 136 |
| Khoudri, I., et al.^76^ | Trend analysis of leprosy in Morocco between 2000 and 2017: Evidence on the single dose rifampicin chemoprophylaxis. | Morocco | 2018 | 801 |
| Post, E., et al.^78^ | Leprosy in The Netherlands in the period 1970-1991. | Netherlands | 1994 | 622 |
| Yu, R., et al.^79^ | Leprosy in New Zealand: An epidemiological update. | New Zealand | 2015 | 38 |
| Al Awaidy, S. T.^81^ | Progress towards a leprosy-free country: the experience of Oman | Oman | 2017 | 77 |
| Goncalves, G., et al.^77^ | Documenting the decline of leprosy in Europe: the example of Northern Portugal | Portugal | 2020 | 15 |
| Assiri, A., et al.^84^ | Eradicating leprosy in Saudi Arabia: Outcome of a ten-year surveillance (2003-2012). | Saudi Arabia | 2014 | 242 |
| Lee, J., et al.^85^ | The decline of leprosy in the Republic of Korea; patterns and trends 1977–2013 | South Korea | 2015 | 24 |
| Serrano-Pozo, A., et al.^89^ | Sensory polyneuropathy as initial manifestation of endemic leprosy in Spain. | Spain | 2004 | 1 |
| Pulido-Perez, A., et al.^90^ | Erythema nodosum leprosum and reversal reaction in 2 cases of imported leprosy. | Spain | 2013 | 2 |
| Norman, F. F., et al.^91^ | Imported and autochthonous leprosy presenting in Madrid (1989–2015): A case series and review of the literature. | Spain | 2016 | 25 |
| Andres, M., et al.^92^ | Lepromatous leprosy presenting as an acute polyarthritis in a Colombian immigrant in Spain. | Spain | 2012 | 1 |
| Alcaide-Martín, A., et al.^93^ | Imported leprosy in Spain. | Spain | 2010 | 6 |
| Suarez-Garcia, I., et al.^88^ | The decline of autochthonous leprosy in the Valencia Region of Spain: patterns and trends 1940-2015. | Spain | 2017 | 21 |
| Suarez-Garcia, I., et al.^86^ | Autochthonous leprosy in Spain: has the transmission of *Mycobacterium leprae* stopped? | Spain | 2020 | 41 |
| Huang, W. L., & Jou, R.^96^ | Leprosy in Taiwan, 2002–2011. | Taiwan (Republic of China) | 2014 | 81 |
| Suchonwanit, P., et al.^99^ | Leprosy reaction in Thai population: a 20-year retrospective study. | Thailand | 2015 | 108 |
| Waters, M. F., & Ridley, D. S.^102^ | Tuberculoid relapse in lepromatous leprosy. | United Kingdom | 1990 | 4 |
| Stafford, S. J., & Wilson, R. R.^103^ | An isolated case of leprosy presenting in a migrant worker in Northern Ireland. | United Kingdom | 2006 | 1 |
| Soni, A., et al.^104^ | Tropical rheumatology in a UK District General Hospital: a case report of leprosy presenting as acute vasculitis. | United Kingdom | 2010 | 1 |
| Malin, A. S., et al.^106^ | Leprosy in reaction: A medical emergency. | United Kingdom | 1991 | 3 |
| Dockrell, H. M., et al.^105^ | Possible transmission of *Mycobacterium leprae* in a group of UK leprosy contacts | United Kingdom | 1991 | 1 |
| Breen, D. P., et al.^101^ | Leprosy: A ‘common’ and curable cause of peripheral neuropathy with skin lesions | United Kingdom | 2015 | 1 |
| Woodall, P., et al.^146^ | Hansen disease among Micronesian and Marshallese persons living in the United States. | United States | 2011 | 177 |
| West, B. C., et al.^145^ | Leprosy in six isolated residents of northern Louisiana. Time-clustered cases in an essentially nonendemic area. | United States | 1988 | 6 |
| Wathen, P. I.^147^ | Hansen's disease. | United States | 1996 | 2 |
| Sotiriou, M. C., et al.^120^ | Case report: Two cases of leprosy in siblings caused by *Mycobacterium lepromatosis* and review of the literature. | United States | 2016 | 2 |
| Sopirala, M. M., et al.^116^ | Acute leprosy in Ohio during treatment of HIV-AIDS. | United States | 2011 | 1 |
| Scollard, D. M., et al.^117^ | Hansen's disease (leprosy) complicated by secondary mycobacterial infection. | United States | 2011 | 1 |
| Scollard, D. M., et al.^115^ | Development of leprosy and type 1 leprosy reactions after treatment with infliximab: a report of 2 cases | United States | 2006 | 2 |
| Schneider, J., & Mays, R.^119^ | A 13-Year-Old with Coexistence of Gastric Volvulus and Leprosy: A Case Report of Two Rare Entities. | United States | 2018 | 1 |
| McKinley, L., & Miller, R.^121^ | A case report and rare side effect of low-dose clofazimine in the treatment of lepromatous leprosy. | United States | 2011 | 1 |
| Martiniuk, F., et al.^118^ | Leprosy as immune reconstitution inflammatory syndrome in HIV-positive persons | United States | 2007 | 2 |
| Marcos, L. A., et al.^122^ | Images in clinical tropical medicine leprosy. | United States | 2014 | 1 |
| Mangum, L., et al.^124^ | Tuberculosis and leprosy coinfection: A perspective on diagnosis and treatment | United States | 2018 | 1 |
| Lyde, C. B.^126^ | Pregnancy in patients with Hansen disease. | United States | 1997 | 3 |
| Lumpkin, I. L. R., et al.^113^ | Leprosy in five armadillo handlers. | United States | 1983 | 5 |
| Lu, P. D., et al.^123^ | HIV and leprosy in the Eastern United States | United States | 2005 | 1 |
| Loh, E., et al.^125^ | Lepromatous leprosy in a 26-year-old man with concurrent disseminated tuberculosis. | United States | 2012 | 1 |
| Logas, C. M., & Holloway, K. B.^127^ | Cutaneous leprosy in Central Florida man with significant armadillo exposure | United States | 2019 | 1 |
| Levis, W. R., et al.^112^ | Leprosy in the eastern united states | United States | 2000 | 2 |
| Levis, W. R., et al.^128^ | Endemic leprosy in New York City. | United States | 2011 | 1 |
| Leon, K. E., et al.^129^ | Case report: Complex type 2 reactions in three patients with Hansen’s disease from a southern United States clinic. | United States | 2015 | 3 |
| Lane, J. E., et al.^131^ | Borderline tuberculoid leprosy in a woman from the state of Georgia with armadillo exposure. | United States | 2006 | 1 |
| Labuda, S., & Stryjewska, B.^130^ | Hansen's disease and rheumatoid arthritis crossover of clinical symptoms: A case series of 18 patients in the united states. | United States | 2016 | 18 |
| Keo, T., et al.^132^ | Molecular origin of endemic leprosy in New York City. | United States | 2008 | 1 |
| Chandranesan, A. S. J., et al.^133^ | Leprosy in Northwest Louisiana: A case series. | United States | 2018 | 5 |
| Hu, S. W.,et al.^149^ | Minocycline-induced hyperpigmentation in multibacillary leprosy. | United States | 2012 | 2 |
| Hartzell, J. D., et al.^110^ | Leprosy: A case series and review. | United States | 2004 | 3 |
| Harb, J., et al.^114^ | Two Cases of Lepromatous Leprosy from Exposure to Armadillos in Florida. | United States | 2017 | 2 |
| Han, X. Y., et al.^3^ | A new Mycobacterium species causing diffuse lepromatous leprosy. | United States | 2008 | 2 |
| Han, X. Y., & Jessurun, J.^139^ | Severe leprosy reactions due to *Mycobacterium lepromatosis*. | United States | 2013 | 1 |
| Gimovsky, A. C., & Macri, C. J.^135^ | Leprosy in pregnant woman, United States. | United States | 2013 | 1 |
| Domozych, R., et al.^136^ | Increasing incidence of leprosy and transmission from armadillos in Central Florida: A case series. | United States | 2016 | 4 |
| Clark, B. M., et al.^137^ | Case-control study of armadillo contact and Hansen's disease. | United States | 2008 | 28 |
| Burdick, A. E., & Ramirez, C. C.^138^ | The role of mycophenolate mofetil in the treatment of leprosy reactions. | United States | 2005 | 3 |
| Breza Jr, T. S., et al.^140^ | False-positive amplified mycobacterium tuberculosis direct tests in skin specimens of leprosy. | United States | 2011 | 2 |
| Aslam, S., et al.^141^ | Major risk factors for leprosy in a non-endemic area of the United States: A case series. | United States | 2019 | 3 |
| Anderson, K. L., et al.^144^ | A case of leprosy in central Florida | United States | 2017 | 1 |
| Abide, J. M., et al.^111^ | Three indigenous cases of leprosy in the Mississippi delta. | United States | 2008 | 3 |
| Bezalel, S., et al.^142^ | Review of leprosy diagnosed at a Midwestern dermatology department, with highlight of *Mycobacterium lepromatosis* in a U.S.-born Midwestern citizen. | United States | 2018 | 9 |
| Williams, D. L., et al.^143^ | Primary multidrug-resistant leprosy, United States. | United States | 2013 | 1 |
| Khang, T. H., & Thao, N. M.^151^ | Epidemiology of Leprosy in Vietnam and the Effectiveness of Multidrug Therapy (MDT) in the Management of the Disease. | Vietnam | 2019 | 96 |
